# Dynamic Evaluation of Circulating miRNA Profile in *EGFR*-Mutated NSCLC Patients Treated with EGFR-TKIs

**DOI:** 10.3390/cells10061520

**Published:** 2021-06-16

**Authors:** Alessandro Leonetti, Mjriam Capula, Roberta Minari, Giulia Mazzaschi, Alessandro Gregori, Btissame El Hassouni, Filippo Papini, Paola Bordi, Michela Verzè, Amir Avan, Marcello Tiseo, Elisa Giovannetti

**Affiliations:** 1 Department of Medicine and Surgery, University of Parma, 43126 Parma, Italy; aleonetti@ao.pr.it; 2 Medical Oncology Unit, University Hospital of Parma, 43126 Parma, Italy; rominari@ao.pr.it (R.M.); giulia.mazzaschi@studenti.unipr.it (G.M.); pbordi@ao.pr.it (P.B.); mverze@ao.pr.it (M.V.); 3 Department of Medical Oncology, Amsterdam UMC, VU University, Cancer Center Amsterdam, 1081 HV Amsterdam, The Netherlands; m.capula@fpscience.it (M.C.); a.gregori@amsterdamumc.nl (A.G.); b.elhassouni@amsterdamumc.nl (B.E.H.); f.papini@student.vu.nl (F.P.); AvanA@mums.ac.ir (A.A.); 4 Fondazione Pisana per la Scienza, San Giuliano, 56017 Pisa, Italy; 5 Institute of Life Sciences, Sant’Anna School of Advanced Studies, 56100 Pisa, Italy; 6 Metabolic Syndrome Research Center, Mashhad University of Medical Sciences, 13131-99137 Mashhad, Iran

**Keywords:** miRNA, *EGFR*-mutated NSCLC, response to EGFR-TKI, targeted therapy

## Abstract

Background: Resistance to EGFR-TKIs constitutes a major challenge for the management of *EGFR*-mutated NSCLC, and recent evidence suggests that deregulation of specific microRNAs (miRNAs) may influence resistance to targeted agents. In this retrospective study, we explored the role of specific plasmatic miRNAs (miR-21, miR-27a and miR-181a) as a surrogate for predicting EGFR-TKI performance in *EGFR*-mutated NSCLC patients. Methods: Plasma samples of 39 advanced *EGFR*-mutated NSCLC patients treated with EGFR-TKIs were collected at different points in time and miRNA levels were assessed by RT-PCR. Results: Higher basal values of miR-21 were reported in patients who achieved a partial/complete response (PR/CR) compared to those with stability/progression of disease (SD/PD) (*p* = 0.011). Along the same line, patients who experienced a clinical benefit lasting at least six months displayed higher basal levels of circulating miR-21 (*p* = 0.039). However, dynamic evaluation of miRNA values after two months from the start of EGFR-TKI treatment showed that patients who experienced SD had an increase in miR-21 levels (Fold Change [FC] = 2.6) compared to patients achieving PR/CR (*p* = 0.029). The same tendency was observed for miR-27a (FC = 3.1) and miR-181a (FC = 2.0), although without reaching statistical significance. Remarkably, preclinical studies showed an increase in miR-21 levels in NSCLC cells that became resistant after exposure to EGFR-TKIs. Conclusions: Our study provides interesting insights on the role of circulating miRNAs, in particular miR-21, and their dynamic change over time in predicting EGFR-TKI response in *EGFR*-mutated NSCLC.

## 1. Introduction

Lung cancer is the second most common malignancy and represents the leading cause of cancer-related death worldwide [1]. Targeted therapy revolutionized the treatment paradigm of advanced non-small-cell lung cancer (NSCLC) in the presence of druggable driver mutations, achieving outstanding results in selected populations. Among driver targetable mutations, Epidermal Growth Factor Receptor (*EGFR*) mutations can be found in approximately 10–16% of NSCLC patients from Western countries, and this percentage is even larger when only considering Asian patients (up to 50%) [2]. 

In the presence of a sensitizing *EGFR* mutation, advanced NSCLC patients are treated frontline with selective tyrosine kinase inhibitors (TKI), among which gefitinib and erlotinib are first-generation TKIs, whereas afatinib and dacomitinib are second-generation irreversible inhibitors. However, despite initial high responses to these drugs, the tumor often implements mechanisms to escape the pathway blockade, ultimately resulting in disease progression [3]. Moreover, given the fact that primary resistance to EGFR-TKIs exists in a portion of patients in the presence of a sensitizing *EGFR* mutation, it is critical to detect potential biomarkers that can help identify the subgroup of patients with primary resistance to EGFR-TKIs therapy. 

Over 50% of acquired resistance to first- and second-generation TKIs is caused by the onset of ‘gatekeeper’ mutation T790M, which compromises the binding of the above-mentioned compounds to EGFR, and increases the receptor affinity for ATP [4]. Third-generation TKI osimertinib can overcome this resistance mechanism, and it has proven to be effective in *EGFR*-mutated NSCLC irrespective of T790M status, but the clinical benefit would be limited by further emergence of resistance [5,6]. Hence, to date, earlier identification of resistance mechanisms as well as the development of new strategies to get through the limitations of the EGFR blockade alone are needed.

MicroRNAs (miRNAs) are 18–25 nucleotides in length, single-stranded non-coding RNAs that function as post-transcriptional regulators of gene expression. Overall, gene silencing mediated by miRNAs is reflected in the regulation of different cellular processes, including cell differentiation, proliferation, apoptosis, and stem cell self-renewal. With respect to carcinogenesis, miRNAs can act as either oncogenes or tumor suppressors, depending on the cellular context and the multiple target genes affected by miRNA silencing [7]. Moreover, accumulating evidence suggests that deregulation of specific miRNAs may also influence cancer cell resistance to conventional chemotherapy and novel targeted agents. 

Regarding *EGFR*-mutated NSCLC, recent evidence suggests that key miRNAs can deregulate pivotal pathways involved in cell survival, metabolism, epithelial-to-mesenchymal transition (EMT), and apoptosis. More interestingly, studies denoted miRNAs as modulators of response to TKIs in *EGFR*-mutated NSCLC, both in vitro and in vivo, thus conditioning both primary and acquired resistance [8]. Furthermore, miRNA expression profiles could serve as biomarkers for predicting patients’ prognosis and response to the targeted treatments. Indeed, growing evidence suggests that dynamic evaluation of miRNA levels, detected in patients’ plasma samples, could represent an emerging useful tool for the monitoring of EGFR-TKI therapy in *EGFR*-driven NSCLC, but this approach has not yet been implemented in the clinical practice [9].

Among different miRNAs, different studies have pointed out miR-21, miR-27a, and miR-181a as potentially responsible for resistance to EGFR-TKIs in *EGFR*-mutated NSCLC. In particular, miR-21 and miR-27a were found to be significantly overexpressed in plasma samples of *EGFR*-mutated NSCLC patients with primary resistance to TKIs compared to the sensitive group [10]. In addition, miR-181a contributed to gefitinib resistance in lung cancer cells by targeting *GAS7*, and was upregulated in gefitinib-resistant cells compared to gefitinib-sensitive cells [11]. Given the preclinical and clinical evidence, a deeper understanding of the clinical implications of miR-21, miR-27a, and miR-181a is warranted. Furthermore, a dynamic evaluation of these miRNAs in blood samples of *EGFR*-mutated patients undergoing TKIs is lacking. Hence, we designed a research study to explore the role of candidate plasma miRNAs (miR-21, miR-27a, and miR-181a) as a surrogate for predicting EGFR-TKIs performance in advanced *EGFR*-mutated NSCLC patients (DynaMiR Study). 

## 2. Materials and Methods

### 2.1. Patients Population

The present research was performed on aliquots of plasma samples that were collected during a previously approved research protocol (DiNAmic study; protocol Version 1, 28 February 2015) at the Department of Medical Oncology of the University Hospital of Parma. Patients must have received EGFR-TKI treatment (gefitinib, erlotinib, or afatinib) for advanced *EGFR*-mutated NSCLC. Only patients with sensitizing *EGFR* mutations were enrolled; patients with non-sensitizing *EGFR* mutations (i.e., ins20) were excluded.

Patients enrolled in the study underwent a blood sample collection at different points in time. A specific consent form for the collection of blood samples was presented to each patient at the time of enrolment into the DiNAmic study (before any specific procedure) and was signed by the patients who decided to take part in the study. 

The following clinico-pathological information was collected at baseline as per clinical practice: age, ECOG PS, comorbidities of the patients, smoking history, number and type of metastatic sites, and presence of brain metastases. Plasma analyses were conducted at the Laboratory Medical Oncology at the Amsterdam University Medical Centers (Amsterdam UMC) VU University, Amsterdam, The Netherlands; and at the Fondazione Pisana per la Scienza, Pisa, Italy.

### 2.2. Plasma Collection and RNA Extraction

Patients underwent collection of 6 mL of blood in Ethylenediaminetetraacetic acid (EDTA) tubes. Samples were collected at the beginning of TKI treatment (t0); after two months (t1); at the time of first radiological evaluation (t2); and at the time of radiologically documented progression of disease, according to RECIST 1.1 criteria (tPD). Tubes were centrifuged twice for 10 min at 2000 rcf within 2 h after collection, and separated plasma was frozen at −80 °C until RNA extraction. 

RNA was extracted from 200 μL plasma using miRNeasy Serum/Plasma Kit (Qiagen, Hilden, Germany) according to the manufacturer’s instructions, with a minor modification: 1 μL of miRNeasy Serum/Plasma Spike-in control (20 fmol cel-miR-39-3p synthetic RNA Spike-In) was added before the addition of Buffer RPL and 1.25 μL of MS2 bacteriophage carrier RNA (Roche, Castle Hill, NSW, Australia). 

### 2.3. miRNA Quantification

RNA samples were reverse-transcribed to cDNA using a cDNA synthesis kit (Qiagen, Hilden, Germany) according to the manufacturer´s instructions. The miRCURY LNA SYBR Green PCR Kit and RT primers (hsa-miR-21-5p, Cat. ID: YP00204230; hsa-miR-27a-3p, Cat. ID: YP00206038; hsa-miR-181a-5p, Cat. ID: YP00206081; and cel-miR-39-3p, Cat. ID: YP00203952), were used for RT-qPCR on CFX96 Real-time System (Biorad, Hercules, CA, USA), in line with the accompanying protocols. 

Cel-miR-39-3p was used as a spike-in control to normalize the variation in RNA extraction, and also as a reference for the relative quantification instead of internal controls. Cyclee thresholds (Cts) were automatically calculated with CFS Manager Software (Biorad, Hercules, CA, USA). 

Technical duplicates were performed for all miRNAs. MiRNA expression was normalized by subtracting the average Ct value of the cel-miR39 from the average Ct value of the miRNA of interest, to obtain the ∆Ct. The miRNA value was expressed as 2^−∆Ct^. Fold-change (FC) was expressed as 2^−∆∆Ct^, according to the ∆∆Ct method [12]. As consistent with previous reports, FC ≥ 2 denoted an increase in miRNA expression, FC ≤ 0.5 denoted a decrease in miRNA expression, and 0.5 < FC < 2 denoted a stability in miRNA expression [13,14].

### 2.4. Cell Culture

The human NSCLC cell lines A549, NCI-H1299, NCI-H23, NCI-H3255, NCI-H1650, and HCC-827 were purchased from ATCC (Manassas, VA, USA) and cultured as recommended. HCC-827GR5 and PC9 were a kind gift from Dr. Pasi A. Jänne, Harvard University, Boston, MA [15]. Cells were grown as a monolayer in 75 cm^2^ flasks (Costar, Cambridge, MA, USA) at 37 °C in 5% CO_2_ and 95% air. The NSCLC cell lines used in this study had been previously characterized for *EGFR* and *KRAS* mutations, and were tested for their authentication by PCR profiling using short tandem repeats (STR), at BaseClear (Leiden, The Netherlands). 

### 2.5. Inhibition of Cell Proliferation

Cell growth inhibition was assessed by a sulforhodamine B (SRB) assay according to the NCI protocol, as described previously [16]. Cells were plated at 10^4^ cells/well, and each drug (gefitinib or afatinib) was tested in triplicate. Growth inhibition was expressed as the percentage of control (0.1% DMSO-treated cells) absorbance (corrected for absorbance before drug exposure). A volume of 25 μL of ice-cold 50% (*w/v*) trichloroacetic acid was added after 72 h treatment. The 50% inhibitory concentration of cell growth (IC50) was calculated by non-linear least squares curve fitting (GraphPad PRISM, Intuitive Software for Science, San Diego, CA, USA).

### 2.6. Establishment and Genetic Characterization of Gefitinib and Afatinib-Resistant Cells

In vitro acquired resistance to gefitinib and afatinib was modeled by applying dose-escalation (up to 1 µM) of these compounds to PC9 cells. Afatinib resistant cells were exposed to a continuous (PC9-AR1) or a pulse (PC9-AR2) exposure. Genomic DNA was extracted using the Ambion^®^-RecoverAll kit (Life Technologies, Breda, The Netherlands). The quantity and purity of the extracted DNA were assessed at 260–280 nm with the NanoDrop^®^-1000-Detector (NanoDrop-Technologies, Wilmington, NC, USA), and *EGFR*, *KRAS*, *BRAF*, and *PIK3CA* mutational statuses were determined as previously described [17]. Nomenclature of EGFR mutations is reported in the Appendix A.

### 2.7. Quantitative PCR Analysis of miR-21 in NSCLC Cells

RNA was extracted according to the Trizol-chloroform protocol and the miR-21 basal expression, and its possible modulation in cells which became resistant to gefitinib and afatinib was assessed by quantitative PCR using the ΔΔCt method [18].

### 2.8. Effects of miR-21 Transfection in Resistant NSCLC Cells

The effect of miR-21 on the inhibition of cell growth was evaluated by transfecting the NSCLC resistant cells with miR-21 antisense oligonucleotides (anti-miR-21), purchased from Ambion-Applied Biosystems (Assay ID, AM10206), at 30 nM final concentration. Cells were also incubated with miRNA negative controls and FAM-labeled anti-miR (Ambion-Applied Biosystems, Waltham, MA, USA), as described previously [18]. 

### 2.9. EGFR and Akt Phosphorylation Assays

To study whether the expression of miR-21 correlated with EGFR activity, as well as whether resistance to EGFR-TKIs was associated to modulation of EGFR downstream pathways, cells were harvested when they were in exponential growth. After protein extraction from cell pellets, EGFR phosphorylation at the tyrosine residue at position 1173 (EGFR [pY1173]) and Akt phosphorylation at serine residue 473 (Akt [pS473]) were evaluated with specific ELISA assays (BioSource International, Camarillo, CA, USA), and were normalized respectively to the total EGFR, Akt and protein content, as described previously [19].

### 2.10. Statistical Analysis

Statistical analysis was performed using SPSS Statistical Software version 27.0 (SPSS, IBM, USA) and GraphPad Prism Software version 6 (Intuitive Software for Science, San Diego, CA, USA). 

Cq values were expressed as mean values ± SD. For each miRNA of interest at the baseline, patients were divided into two groups with respect to the median expression value of the miRNA (high vs. low). Wilcoxon and Mann–Whitney tests were used to compare the two groups of paired and unpaired data, respectively. The Kruskal–Wallis test was employed for the analysis of group differences. Progression-free survival (PFS) and overall survival (OS) were calculated from the start of EGFR-TKI treatment until radiological confirmed progression of disease or death, respectively, using the Kaplan–Meier method. Statistical differences were assessed using the log-rank test. All *p*-values were two sided, and *p* < 0.05 was used to indicate a statistically significant difference.

All in vitro experiments were performed in triplicate and repeated at least twice. Data were expressed as mean values ± SEM and analyzed using the two-tailed Student’s t-test or ANOVA, followed by the Bonferroni’s multiple comparison test using GraphPad Prism Software version 6 (Intuitive Software for Science, San Diego, CA, USA). The level of significance was *p* < 0.05.

## 3. Results

### 3.1. Patient Characteristics

The study population involved 39 patients. Baseline patient characteristics are summarized in Table 1.

Concerning the correlation between clinico-pathological characteristics and survival outcome, Kaplan–Meier curves showed that patients with brain metastases at baseline had a shorter OS than patients without brain metastases (median OS: 14.0 vs. 37.3 months [Hazard Ratio (HR), 2.49; 95% CI: 1.13–5.48; *p* = 0.023]) (Figure S1A). Along the same line, patients who had liver metastases at baseline displayed shorter OS than patients without liver metastatic involvement (median OS: 11.6 vs. 34.1 months [HR, 3.48; 95% CI: 1.29–9.41; *p* = 0.023], *p* = 0.014) (Figure S1B). None of the other basal features (sex, age, smoking status, number of metastatic sites, and type of *EGFR* mutation) were significantly associated with either PFS or OS. No correlations were observed between baseline patient characteristics and either response to EGFR-TKIs (PR/SD vs. PD) or duration of clinical benefit (≥6 months vs. <6 months) (data not shown).

### 3.2. Basal miRNA Expression and Correlation with Clinical Features

Clinical characteristics of patients (gender, smoking status, bone metastasis, central nervous system metastasis, pleural effusion, liver metastasis, number of metastatic sites, and type of *EGFR* mutation) were subsequently correlated with basal values of miR-21, miR-27a and miR-181a. Among all features, patients aged <65 years had significantly higher basal values of miR-21 compared to the counterpart (*p* = 0.044). No further differences in basal miRNA expression were observed according to other clinical features (Figures S2 and S3). When looking at relevant comorbidities, we did not find any difference in basal miR-21, miR-27a and miR-181a according to the presence/absence of comorbidity (Table S2).

### 3.3. Basal miRNA Expression and Correlation with Clinical Outcome

All the enrolled patients had baseline samples collected before the start of EGFR-TKI treatment. As reported in Figure S4, basal levels of the three candidate miRNAs were significantly correlated to each other (miR-21 vs. miR-27a: R = 0.922, *p* < 0.001; miR-21 vs. miR-181a: R = 0.789, *p* < 0.001; miR-27a vs. miR-181a: R = 0.732, *p* < 0.001). This is in agreement with previous studies showing that miR-21 and miR-27a acted as cooperative repressors of a network of tumor suppressor genes that included PDCD4, BTG2, and NEDD4L [20].

**Figure 1:**
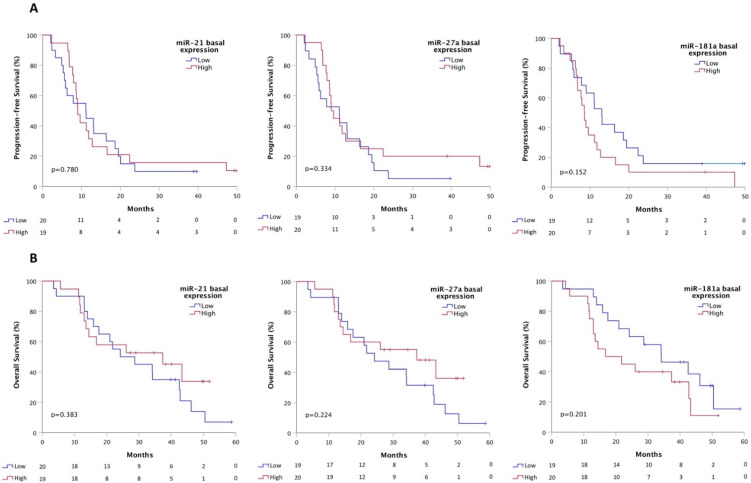
Kaplan–Meier curves for basal miRNA expression and correlation with PFS (**A**) and OS (**B**).

In order to find a putative correlation between basal miRNA values and clinical outcomes, patients were divided into two groups with respect to the median expression value of the investigated miRNA (High vs. Low). Basal values of miR-21, miR-27a and miR-181a were not significantly correlated with PFS (miR-21 Low vs. High: median PFS 11.1 vs. 9.0 months, *p* = 0.780; miR-27a Low vs. High: median PFS 11.1 vs. 9.0 months, *p* = 0.334; miR-181a Low vs. High: median PFS 13.1 vs. 8.5 months, *p* = 0.152) (Figure 1A). The same trend was observed for the OS (miR-21 Low vs. High: median OS 24.2 vs. 37.3 months, *p* = 0.383; miR-27a Low vs. High: median OS 24.2 vs. 37.3 months, *p* = 0.224; miR-181a Low vs. High: median OS 34.1 vs. 16.8 months, *p* = 0.201) (Figure 1B).

**Figure 2:**
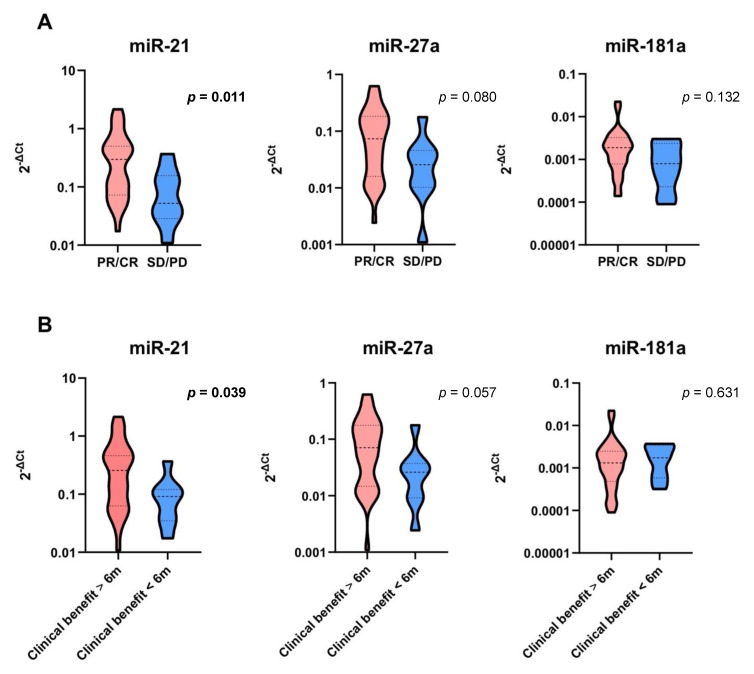
Basal miRNA expression and correlation with EGFR-TKI response (**A**) and duration of clinical benefit (**B**). Significant *p* values are in bold. Dashed lines represent the median values. Dotted lines represent the upper and lower quartiles.

Basal differential expression of the candidate miRNAs was evaluated on the basis of the best objective response to EGFR-TKI treatment. When patients were stratified according to their best response (partial response/complete response [PR/CR] vs. stable disease/progression of disease [SD/PD]), patients who achieved PR/CR as their best response had a significantly higher basal value of miR-21 than patients who achieved SD/PD (*p* = 0.011) (Figure 2A). Dividing the patients according to the duration of clinical benefit (≥6 months vs. <6 months), patients who achieved a clinical benefit ≥6 months had a higher basal value of miR-21 than patients with clinical benefit <6 months (*p* = 0.039) (Figure 2B). 

When patients were stratified according to basal miRNA expression, those who had basal overexpression of either miR-21 or miR-27a were more likely to achieve a durable clinical benefit ≥6 months (Fisher’s exact test *p* = 0.044 and *p* = 0.020, respectively) (Table 2).

### 3.4. Modulation of miRNA Expression in Patients Treated with EGFR-TKIs

Modulation of basal miRNA expression was evaluated on blood samples collected after two months from the beginning of TKI treatment and prior to the first radiological assessment, labeled as ‘t1’, in order to correlate early variations of circulating miRNAs to differential EGFR-TKI responses.

From the enrolled patients, 24 patients (62%) had ‘t1’ samples available for this analysis and, among them, no one experienced PD as best response. Patients were then stratified according to their best response (PR/CR vs. SD) and the duration of clinical benefit (≥6 months vs. <6 months). 

**Figure 3:**
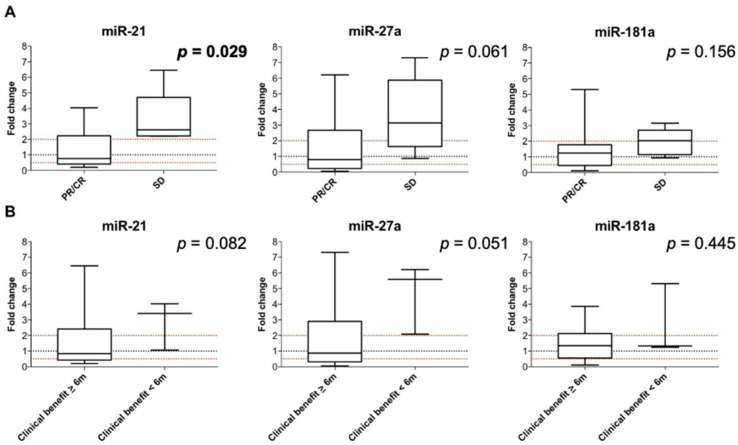
Modulation of miRNA expression at t1 and correlation with EGFR-TKI response (**A**) and duration of clinical benefit (**B**). Significant *p* values are in bold. The box plot represents the upper to lower quartiles, the whiskers are the minimum and maximum values, and the black lines represent the median values.

Statistically significant differences in FC were observed for miR-21 at t1 compared to baseline according to the type of best response to EGFR-TKI (PR/CR vs. SD): miR-21 median FC at t1 in PR/CR vs. SD: 0.8 vs. 2.6, *p* = 0.029. No statistically significant difference in FC was observed for miR27a and miR-181a at t1 compared to baseline according to the type of best response to EGFR-TKI, even though a trend towards an increase in miR-27a and miR-181a at t1 was observed for patients with SD compared to PR/CR: miR-27a median FC at t1 in PR/CR vs. SD: 0.8 vs. 3.1, *p* = 0.061; miR-181a median FC at t1 in PR/CR vs. SD: 1.2 vs. 2.0, *p* = 0.156 (Figure 3A).

No statistically significant difference in FC was observed for miRNAs compared to baseline according to the clinical benefit to EGFR-TKI, even though a trend towards an increase in miR-21 and miR-27a at t1 was observed for patients with clinical benefit <6 months compared to patients with clinical benefit ≥6 months: miR-21 median FC at t1 in clinical benefit ≥6 months vs. <6 months: 0.8 vs. 3.4, *p* = 0.082; miR-27a median FC at t1 in clinical benefit ≥6 months vs. <6 months: 0.9 vs. 5.6, *p* = 0.051; miR-181a median FC at t1 in clinical benefit ≥6 months vs. <6 months: 1.3 vs. 1.3, *p* = 0.445 (Figure 3B). 

Since an increase in miRNAs at t1 denoted patients who were likely to achieve SD as their best response rather than PR, as well as a limited clinical benefit, patients were further stratified according to the increase (FC ≥ 2) vs. decrease/stability (FC < 2) of miRNAs at t1, and groups were compared in terms of PFS and OS. No statistically significant differences were observed between patients who had an increase in miRNAs compared to those who had a decrease/stability at t1, both in terms of PFS and OS (data not shown). 

### 3.5. Modulation of miRNA Expression at the Time of First Radiological Evaluation

Change in basal miRNA expression was evaluated on blood samples collected at the time of first radiological assessment, labeled as ‘t2’. Thirty-two patients (82%) had ‘t2’ samples available for this analysis. Patients were stratified according to their best response (PR/CR vs. SD/PD) and the duration of clinical benefit (≥6 months vs. <6 months). 

No statistically significant difference in FC was observed for miRNAs at t2 compared to baseline according to both the type of best response and clinical benefit to EGFR-TKI (Figure S5). No statistically significant differences were observed between patients who had an increase in miRNAs compared to those who had a decrease/stability at t2, both in terms of PFS and OS (data not shown).

### 3.6. Modulation of miRNA Expression at the Time of Progression of Disease

**Figure 4:**
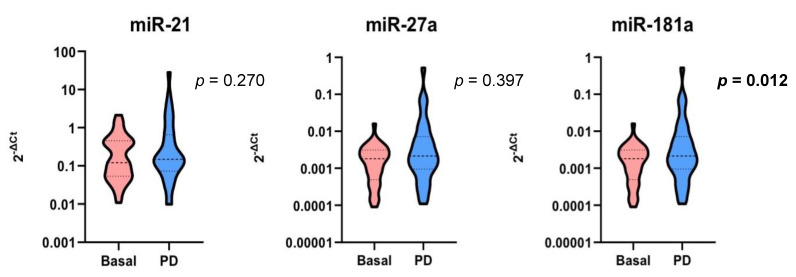
Modulation of miRNA expression at the time of progression to EGFR-TKI compared to baseline. Significant *p* values are in bold. Dashed lines represent the median values. Dotted lines represent the upper and lower quartiles.

Change from basal miRNA expression was evaluated from blood samples collected at the time of progression to EGFR-TKI treatment, labeled as ‘tPD’. Among the 35 patients who experienced disease progression, one patient did not have a tPD sample available and was excluded from this analysis. miR-181a levels at the time of progression were stable compared to the basal values, and this data was statistically significant (median FC: 1.7, *p* = 0.012). miR-21 and miR-27a levels were stable compared to the relative basal values, although without reaching statistical significance (median FC miR-21: 1.1, *p* = 0.270; median FC miR-27a: 1.3, *p* = 0.397, respectively) (Figure 4).

### 3.7. Correlation between miR-21 Expression, Chemosensitivity and Phosphorylated-EGFR Levels in NSCLC Cell Lines

The expression of miR-21 showed a large heterogeneity, and we evaluated whether the different sensitivity to drug treatment may be related to variable cellular miR-21 expression profiles (Table S1) and to phospho-EGFR in a panel of NSCLC cells characterized by their *EGFR* status.

**Figure 5:**
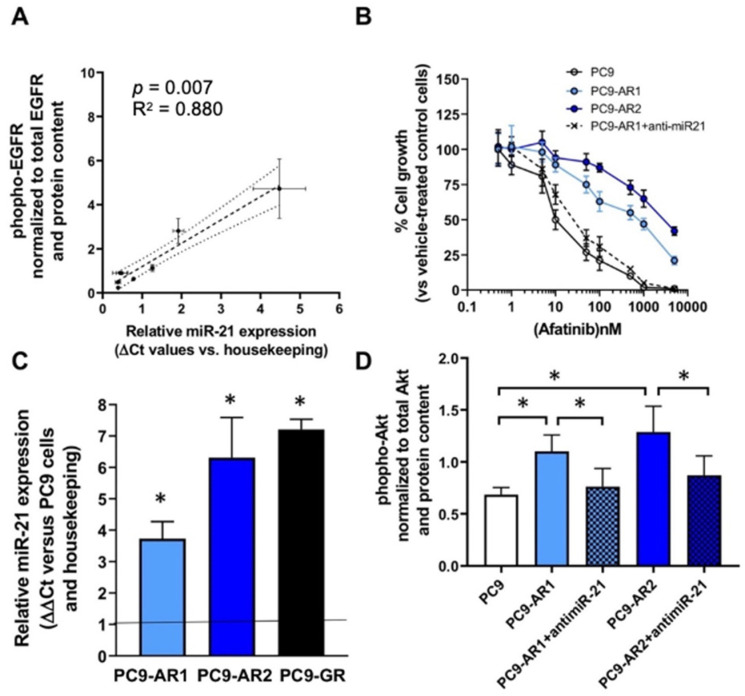
In vitro studies on the impact of miR-21 on phospho-EGFR and resistance to EGFR-TKIs: (**A**) Spearman correlation between miR-21 expression and phospho-EGFR levels in A549, NCI-H1299, NCI-H1703, NCI-H23, NCI-H1650, HCC827GR5, NCI-H3255, HCC827 and PC9 cells; (**B**) Inhibition of cell growth by afatinib in PC9 cells and the resistant cells PC9-AR1, PC9- AR2 and PC9-AR1 cells transfected with anti-miR-21; (**C**) Modulation of miR-21 expression in PC9 cells resistant to afatinib and gefitinib (*p* < 0.05 compared to wild type cells); (**D**) Modulation of phospho-Akt expression in PC9 cells resistant to afatinib and gefitinib cells transfected with anti-miR-21 or scramble negative controls (* *p* < 0.05).

The cells harboring *EGFR* activating mutations (NCI-H3255, HCC-827 and PC9) were the most sensitive to all the EGFR-TKIs, and had the highest expression levels of both miR-21 and phospho-EGFR (Figure 5A). 

Conversely, the cells harboring both *EGFR* mutations and *PTEN* exon-9 loss or *cMET* amplification (i.e., NCI-H1650 and HCC827 cells) had IC_50_ and miR-21 values similar to the cells with wild-type *EGFR* status. No correlation was found between *KRAS* mutations and either miR-21 expression or drug sensitivity.

Next, we evaluated the modulation of miR-21 expression in the clones selected for their resistance to gefitinib and afatinib. After approximately six months, resistant clones emerged which were over 100-fold less sensitive to the drug (Figure 5B) than the parental cell line. Melting PCR and sequencing analyses were performed to select clones which did not have additional mutations in *EGFR* (exons 18–21), *KRAS* (exons 2–3), *BRAF* (exon 15) and *PIK3CA* (exons 9 and 20) genes. Interestingly, in all these clones, we observed a significant increase of miR-21 (of 2.7–3.8-fold, *p* < 0.05), as reported in Figure 5C.

In order to further explore the role of miR-21 in the sensitivity of resistant cells, the PC9-AR1 cells were transfected with miR-21-specific antisense inhibitors. Transfection efficiency was evaluated by analysis of fluorescent microscope images of cells transfected 24 h before with specific FAM-dye anti-miR oligonucleotides, showing at least 70% transfection efficiency, with >90% cell viability. Furthermore, quantitative PCR analysis of miR-21 after anti-miR21 transfection showed a 2-fold reduction of miR-21 expression compared to cells transduced with negative controls (data not shown). The reduced expression of miR-21 was associated with a partial rescue of the sensitivity to afatinib, with more than 50-fold reduction of the IC50 in the PC9-AR1, suggesting that the aberrant expression of this miRNA correlated to drug resistance (Figure 5B). Similar results were observed for PC9-AR2 cells (data not shown).

To investigate the effects of miR-21 on EGFR downstream pathways, we evaluated the phosphorylation status of Akt in the wild-type/control, resistant and anti-miR21 transfected cells. The resistant cells were characterized by a significant increase of the phopho-Akt/Akt ratio ranging from +61 to +88% in the PC9-AR1 and PC9-AR2 cells, respectively. In contrast, transfection with anti-miR-21 significantly reduced the activation status of Akt, with a pAkt/Akt ratio from 1.101 to 0.763 U/ng in PC9-AR1 and from 1.289 to 0.872 U/ng in PC9-AR2 cells (Figure 5D).

## 4. Discussion

In the present study, three candidate circulating miRNAs (miR-21, miR-27a, and miR-181a) were evaluated as prognostic and predictive factors of response to EGFR-TKI treatment in 39 advanced *EGFR*-mutated NSCLC patients who underwent EGFR-TKIs. 

Firstly, we evaluated the correlation of basal miRNA expression with clinical characteristics of patients, thus documenting an inverse association between values of miR-21 and age, as previously reported [21]. 

Next, we explored the correlation of basal miRNA expression, defined as the level of circulating miRNA prior to EGFR-TKI, with survival outcomes and treatment response. Lacking a control group of matched healthy volunteers, high/low basal miRNA expression was established based on the median value for each miRNA. We did not find any statistically significant impact of basal miRNA expression on PFS and OS. However, higher basal values of miR-21 were documented in patients who achieved PR/CR as best response compared to those with SD/PD (*p* = 0.011). Along the same line, patients who experienced a clinical benefit lasting at least six months displayed higher levels of circulating miR-21 (*p* = 0.039). 

Dynamic variations of miRNA values after two months from the start of EGFR-TKI treatment (t1) were assessed to explore an early change of circulating miRNA expression as a way to predict differential EGFR-TKI response. Of note, at this time point, patients who experienced SD had an increase in miR-21 levels (FC = 2.6) compared to patients achieving a response (PR/CR) (*p* = 0.029). The same tendency was observed for miR-27a (FC = 3.1) and miR-181a (FC = 2.0), although without reaching statistical significance.

However, when focusing on circulating levels of the candidate miRNAs at the time of the first radiological evaluation, we did not find any relevant difference in FC from basal. This evidence suggests that an early assessment of circulating miR-21 (t1) could be more informative for predicting the response to EGFR-TKIs than that performed at the time of the first radiological examination. 

Similarly, no differences were found for miR-21, miR-27a, and miR-181a at the time of progression to EGFR-TKIs. In contrast, miR-21 was upregulated in gefitinib- and afatinib-resistant NSCLC cells. In this regard, we could not exclude that the type of resistance mechanism to EGFR-TKI could have an impact on the levels of the candidate miRNAs, as most of our patients developed T790M resistance mutation at the time of PD, whereas cell lines harbored an EGFR-independent mechanism of resistance. Our preclinical findings are in line with the results of a recent study, showing that miR-21 was overexpressed in PC9 gefitinib-resistant (PC9R) cells that still had the *EGFR* mutation, but no T790M mutation [22]. Moreover, T790M *EGFR*-mutated NSCLC showed a different miRNA profile than tumors harboring L858R-activating mutations [23], suggesting a different role of miRNA expression in the resistance mechanisms in NSCLC cells harboring different *EGFR* mutations.

To date, this is the first study aimed at investigating the dynamic modification of circulating plasma miRNA levels as a predictor of response to different EGFR-TKIs (gefitinib, erlotinib, and afatinib) in advanced *EGFR*-mutated NSCLC. Previous research suggested that miR-21, miR-27a, and miR-181a may act as tumor-promoting miRNAs in NSCLC, and an increase in their circulating values could be associated with EGFR-TKI resistance [10,11,22,24]. The oncogenic properties of miR-21 in NSCLC can be explained by miR-21 target genes, which are involved in multiple pathways such as cell growth and proliferation, angiogenesis, invasion and metastasis, as also reported in recent extensive reviews and meta-analyses [24,25,26]. In particular, we demonstrated that the increase in miR-21 levels in our resistant cell lines was associated with increased phosphorylation of Akt. Conversely, downregulation of miR-21 resulted in less active signaling through the PI3-kinase-Akt pathway, rendering the cancer cells more susceptible to drug activity. Previous studies showed that miR-21 does indeed regulate the expression of PTEN and phosphorylation of its downstream kinase PI3-kinase-Akt pathway [27,28,29,30,31]. Moreover, the increase of phospho-Akt correlated with the reduction of drug-induced apoptosis and antitumor activity, suggesting that the Akt pathway plays a significant role in mediating drug resistance in different cancer cell models [30,32]. 

Our results are in line with these pieces of evidence, as an increase in miR-21 at t1 was associated with a lack of response to EGFR-TKIs, here intended as stability of disease. Accordingly, a trend of increased miR-27a and miR-181a in SD patients at t1 was apparent in our cohort, thus underlying their oncogenic properties. Of note, miR-21 is a well-known oncomiR in different tumors, and we demonstrated that miR-21 and miR-27 cooperatively inhibit a network of tumor suppressor genes involved with pancreatic tumor growth and progression [19]. Furthermore, previous studies suggested that plasma levels of miR-21 and miR-181 reflected tissue expression [33,34,35], and PCR-based analysis of pre- and post-chemotherapy plasma samples showed that the downregulation of plasma miR-181a-5p predicted longer survival in pancreatic cancer patients [34].

Our results that high basal miR-21 levels were directly correlated with disease response and clinical benefit seem contradictory, but might be explained by the fact that miR-21 is a downstream effector of the activated EGFR signaling pathway. A statistically significant positive correlation was indeed observed between miR-21 expression levels and phospho-EGFR levels in NSCLC cell lines. These results are in agreement with previous findings in both NSCLC tissues and cell lines [36], and with the hypothesis that activator protein-1, which is activated by EGFR signaling [37], can in turn activate the miR-21 transcription through the binding to the promoter [38]. However, our study shows that miR-21 levels were modulated after treatment, and we hypothesize that a single-time assessment of circulating miRNA could be less adequate to predict treatment outcome compared to dynamic monitoring.

Our study has some limitations that could have influenced our results. First, we lacked a control group for assessing the relative expression of our candidate miRNAs in NSCLC patients compared to healthy individuals. Second, candidate miRNAs were arbitrarily selected based on available literature; miRNA PCR profiling in an initial discovery phase could have more accurately detected deregulated miRNAs. Moreover, our sample size was relatively small and heterogeneous, and this could have limited the detection of statistically significant differences, even considering the exploratory purpose of our research. However, our positive results surely deserve further investigation in a larger prospective study. Furthermore, a spike-in control was used as a reference for the relative miRNA quantification instead of internal controls. Although cel-miR-39 has been extensively used as a normalizer for circulating miRNA quantification, spike-in controls cannot normalize variations caused by factors prior to RNA isolation [39]. In addition, modulation of miR-21 might not only characterize cancer cells, but also be a common feature of pathological cell growth, as observed in mouse models with hypertrophic heart and other non-neoplastic diseases [40,41]. In this sense, we excluded a putative influence of comorbidities on miRNA circulating levels in our cohort of patients. Even though we did not perform concordance analysis between plasma and tissue levels, due to the lack of available material, we could assume that circulating levels reflected those detected in the primary tumor, as already reported [42]. Finally, since the third-generation EGFR-TKI osimertinib replaced early-generation EGFR-TKIs for the frontline treatment of metastatic *EGFR*-mutated NSCLC, our results need to be validated in this clinical setting as well. Nevertheless, the study setup and results can serve as a framework for further studies. 

## 5. Conclusions

This study provides interesting hints about the role of dynamic changes of circulating miRNAs in predicting EGFR-TKI response in advanced *EGFR*-mutated NSCLC. Considering the minimal invasiveness of blood sampling, the evaluation of early circulating miRNA modification, especially miR-21, could indeed represent a useful tool for monitoring treatment outcome. Further extensive and prospective studies are warranted to confirm the predictive role of circulating miRNAs. These predictive biomarkers will then hopefully be applied in the clinical setting to select the best therapeutic approaches, thus circumventing unnecessary treatments and preventing collateral side effects.

## Figures and Tables

**Table 1:** Patient Characteristics.

Patient Characteristics	Number (%)
*Gender*	
Male	16 (41)
Female	23 (59)
*Age at Diagnosis*	
<65 years	18 (46)
≥65 years	21 (54)
*Smoking Status*	
Current/former	18 (46)
Never	21 (54)
*ECOG PS*	
0	21 (54)
1	16 (41)
2	2 (5)
*Brain Mets at Baseline*	
No	24 (61)
Yes	15 (39)
*Liver Mets at Baseline*	
No	34 (87)
Yes	5 (13)
*Bone Mets at Baseline*	
No	24 (61)
Yes	15 (39)
*Number of Metastatic Sites*	
<3	22 (56)
≥3	17 (44)
*EGFR Mutation*	
Ex19del	19 (49)
L858R	16 (41)
G719X + S768I	1 (2.5)
Ex19del + T790M	1 (2.5)
L858R + S768I	1 (2.5)
S768I	1 (2.5)
*EGFR-TKI*	
Gefitinib	23 (59)
Erlotinib	7 (18)
Afatinib	9 (23)
*Line of Treatment*	
First-line	37 (95)
Second-line	2 (5)
*Best Response to EGFR-TKI*	
CR	1 (2.5)
PR	28 (72)
SD	6 (15.5)
PD	4 (10)

**Table 2:** Correlation between basal miRNA values and clinical benefit to EGFR-TKI.

Basal miRNA Expression	Clinical Benefit <6 Months	Clinical Benefit ≥6 Months	*p* Values
miR-21 low (%)	7 (35%)	13 (65%)	0.044
miR-21 high (%)	1 (5%)	18 (95%)	
miR-27a low (%)	7 (37%)	12 (63%)	0.020
miR-27a high (%)	1 (5%)	19 (95%)	
miR-181a low (%)	5 (26%)	14 (74%)	0.451
miR-181a high (%)	3 (15%)	17 (85%)	

## Data Availability

The data presented in this study are available on request from the corresponding author. The data of the patients are not publicly available due to privacy rules.

## Supplementary Materials

The following are available online at https://www.mdpi.com/article/10.3390/cells10061520/s1, Figure S1: Kaplan Meier curves for central nervous system (CNS) metastases (A) and liver metastasis (B) and correlation with OS; Figure S2: Basal miRNA expression and correlation with gender (A), smoking status (B), age (C) and type of EGFR mutation (D); Figure S3. Basal miRNA expression and correlation with bone metastasis (A), central nervous system (CNS) metastasis (B), liver metastasis (C) and number of metastatic sites (D); Figure S4: Correlation between basal miRNA values; Figure S5: Modulation of miRNA expression at t2 and correlation with EGFR-TKI response (A) and duration of clinical benefit (B); Table S1: Anti-proliferative effect of gefitinib, afatinib or osimertinib in NSCLC cells characterized for their EGFR and K-RAS status; Table S2: List of patients’ comorbidities and correlation with miRNA levels.

## Appendix A. HGVS. Nomenclature of *EGFR* Mutations

Exon 18

G719X: c.2155G > A p.(G719S); c.2155G > T p.(G719C); c.2156G > C p.(G719A).

Ex19del: c.2235_2249del15; c.2235_2252 > AAT (complex); c.2236_2253del18; c.2237_2251del15; c.2237_2254del18; c.2237_2255 > T (complex); c.2236_2250del15; c.2238_2255del18; c.2238_2248 > GC (complex); c.2238_2252 > GCA (complex); c.2239_2247del9; c.2239_2253del15; c.2239_2256del18; c.2239_2248TTAAGAGAAG > C(complex); c.2239_2258 > CA (complex); c.2240_2251del12; c.2240_2257del18; c.2240_2254del15; c.2239_2251 > C (complex).

Exon 20

Ins20: c.2307_2308ins9; c.2319_2320insCAC; c.2310_2311insGGT;

S768I: c.2303G > T p.(S768I);

T790M: c.2369C > T p.(T790M).

Exon 21

L858R: c.2573T > G p.(L858R).
