# Supplementary material for: Dynamic Evaluation of Circulating miRNA Profile in EGFR-Mutated NSCLC Patients Treated with EGFR-TKIs
Source: Cells. 2021 Jun 16;10(6):1520. doi: 10.3390/cells10061520 (PMC8235748; doi:10.3390/cells10061520)
Supplement: Supplementary file 1 [file cells-10-01520-s001.zip › cells-1245856-supplementary.pdf]

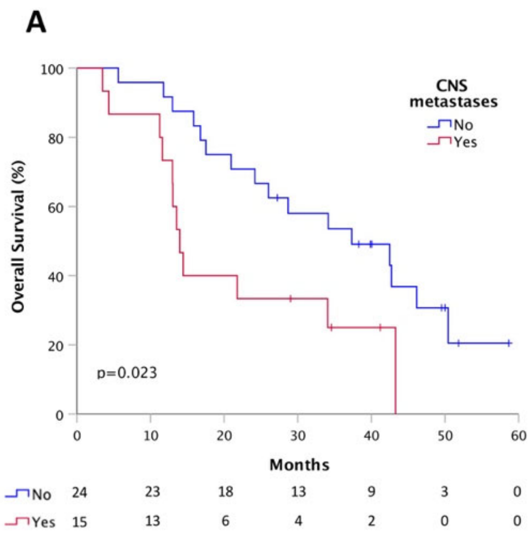

Figure S1. Kaplan Meier curves for central nervous system (CNS) metastases (A) and liver metastasis (B) and correlation with OS.

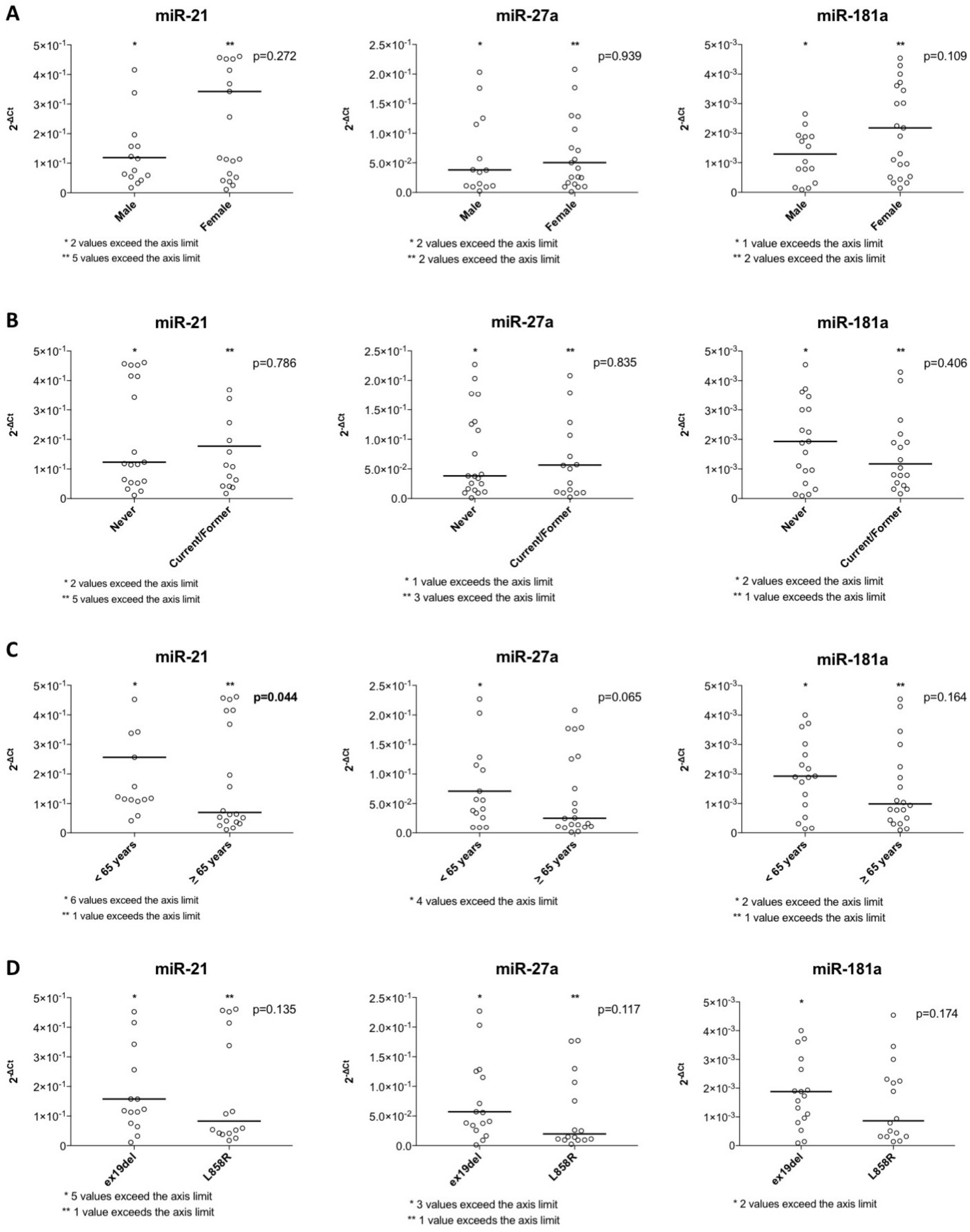

Figure S2. Basal miRNA expression and correlation with gender (A), smoking status (B), age (C) and type of *EGFR* mutation (D). The black line represents the median value in each graph.

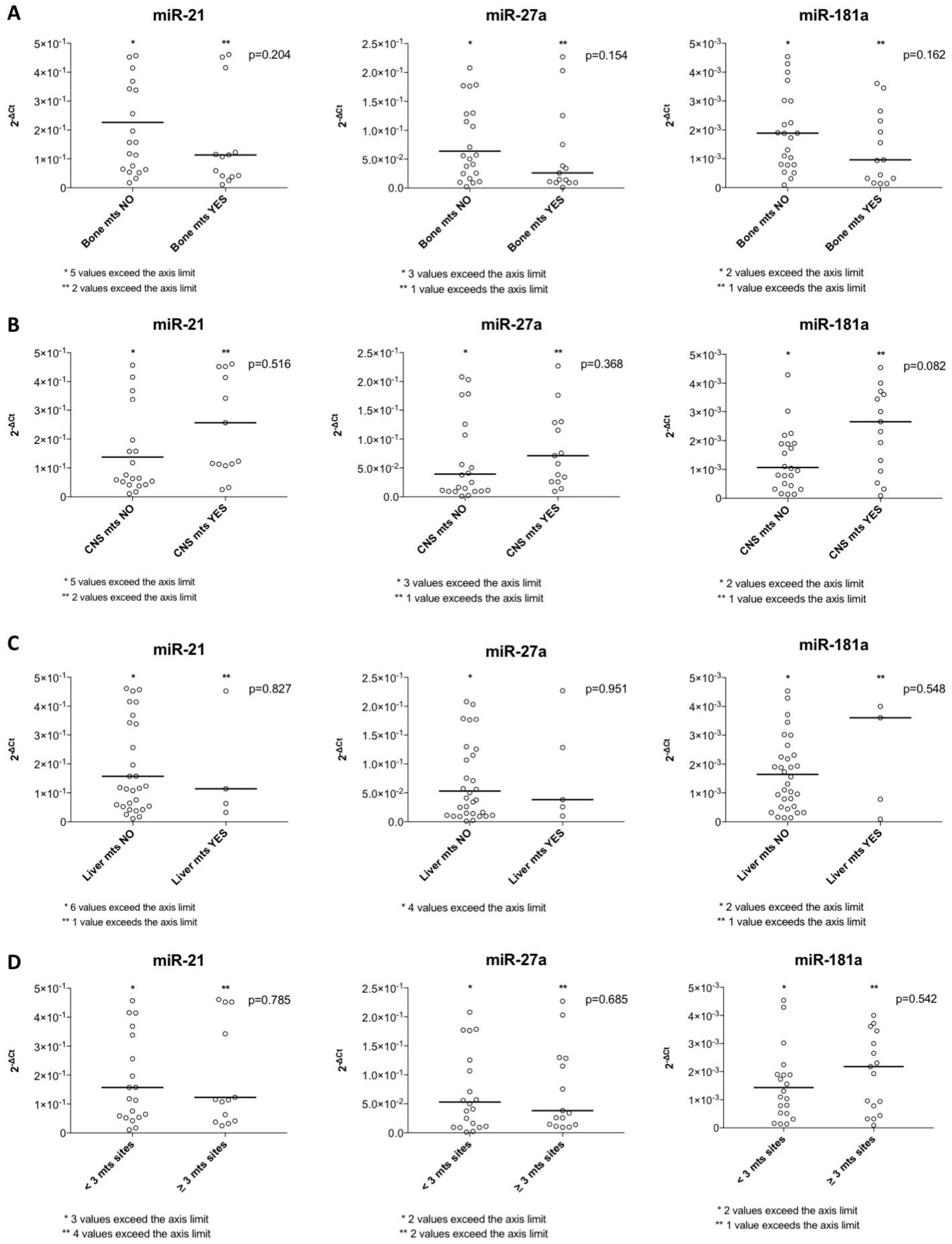

Figure S3. Basal miRNA expression and correlation with bone metastasis (mts) (A), central nervous system (CNS) mts (B), liver mts (C) and number of mts sites (D). The black line represents the median value in each graph.

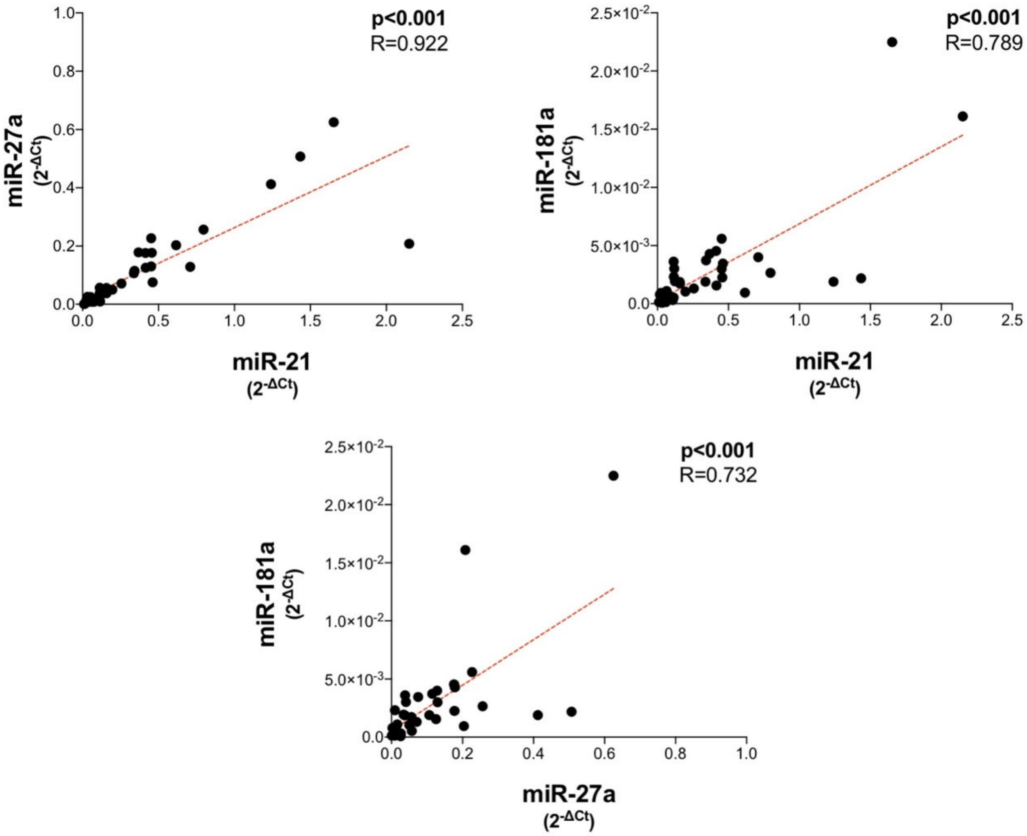

Figure S4. Correlation between basal miRNA values.

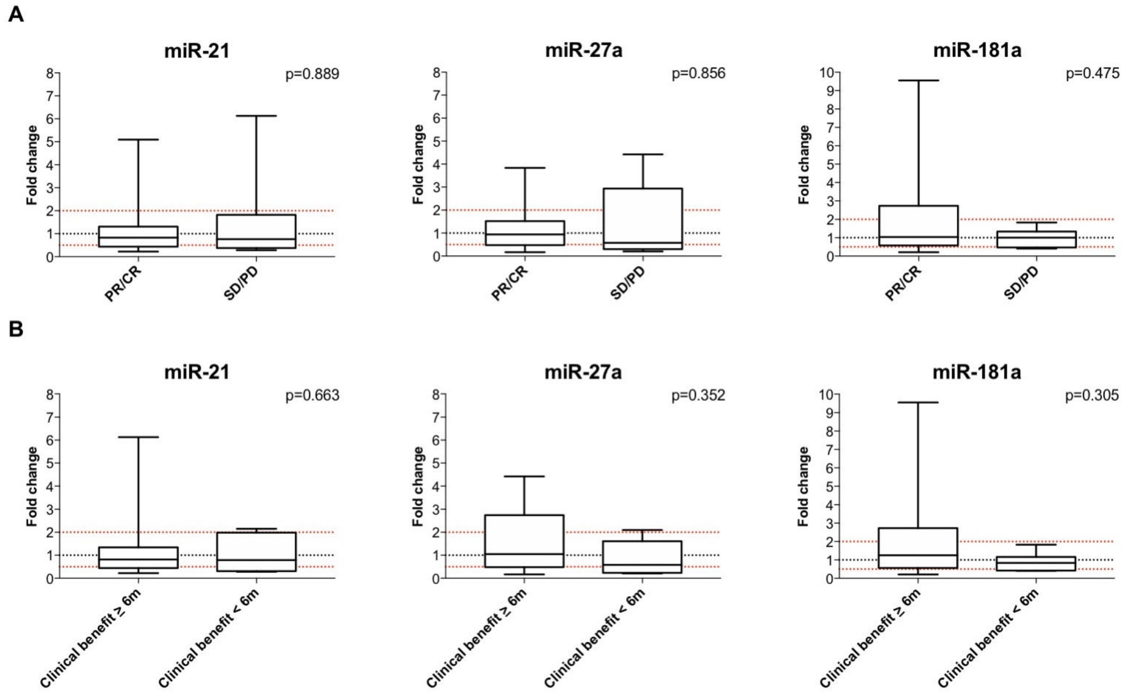

Figure S5. Modulation of miRNA expression at t2 and correlation with EGFR-TKI response (A) and duration of clinical benefit (B).

Table S1. Anti-proliferative effect of gefitinib, afatinib or osimertinib in NSCLC cells characterized for their *EGFR* and *K-RAS* status.

Cell line	*EGFR* status	*KRAS* status	Gefitinib IC_{50} (μM)	Afatinib IC_{50} (μM)
A549	WT	Mut (G12S)	8.0±0.2	5.3±0.1
NCI-H1299	WT	WT	7.9±0.6	5.6±0.4
NCI-H23	WT	Mut (G12C)	11.2±0.7	4.5±0.2
NCI-H1650	delE746-A750 (*PTEN del*)	WT	2.0±0.03	3.3±0.2
HCC827GR5	delE746-A750 (*cMET ampl*)	WT	8.7±0.7	5.0±0.6
NCI-H3255	L858R	WT	0.020±0.004	0.008±0.002
HCC827	delE746-A750	WT	0.011±0.003	0.005±0.001
PC9	delE746-A750	WT	0.040±0.004	0.009±0.001

WT, wild-type; Mut, mutant; del, deletion; ampl, amplification

Table S2. List of patients' comorbidities and correlation with miRNA levels.

Comorbidity	Number of patients (%)	Differences in miR-21	Differences in miR-27a	Differences in miR-181a
Cardiovascular:				
• Hypertension	18 (46)	p=0.995	p=0.549	p=0.651
• Ischemic cardiomyopathy	5 (13)	p=0.665	p=0.223	p=0.420
• Arhythmia	5 (13)	p=0.237	p=0.378	p=0.137
Metabolic:				
• Diabetes	7 (18)	p=0.576	p=0.255	p=0.512
• Hyperlipidemia	5 (13)	p=0.606	p=0.639	p=0.505
Viral hepatitis	3 (13)	p=0.596	p=0.564	p=0.468
